# Biosimilars of anti-VEGF agents in retinal diseases: a narrative review of regulatory, clinical, and pharmacoeconomic aspects

**DOI:** 10.1186/s40942-026-00872-9

**Published:** 2026-05-30

**Authors:** Yuan Zong, Qiwei Fan, Lijun Huang

**Affiliations:** 1 Department of Ophthalmology, The First Affiliated Hospital of Jinan University, Guangzhou, 510630 China; 2 Department of Ophthalmology, Zhongshan Torch Development Zone People’s Hospital, Zhongshan, Guangdong, 528436 China; 3 International Ocular Surface Research Center, Institute of Ophthalmology, and Key Laboratory for Regenerative Medicine, Jinan University Medical School, Guangzhou, 510632 China

**Keywords:** Biosimilars, Anti-VEGF, Ranibizumab, Aflibercept, Bevacizumab, Pharmacoeconomics, Drug delivery systems

## Abstract

**Background:**

Anti-vascular endothelial growth factor (anti-VEGF) agents are the cornerstone therapy for retinal vascular diseases including neovascular age-related macular degeneration, diabetic macular edema, and retinal vein occlusion. With patent expirations of ranibizumab and aflibercept, biosimilars have emerged as cost-effective alternatives. However, vitreoretinal specialists require consolidated evidence on regulatory standards, clinical equivalence, and practical implementation strategies to guide evidence-based integration of biosimilars into clinical practice.

**Methods:**

We conducted a narrative review of regulatory documents from the FDA, EMA, and NMPA, as well as Phase III clinical trials, real-world studies, and pharmacoeconomic analyses of approved ophthalmic anti-VEGF biosimilars. Literature searches were performed across PubMed, Embase, and regulatory databases from 2015 to 2025. Studies were included if they reported regulatory approval pathways, clinical efficacy and safety data, interchangeability studies, or pharmacoeconomic evaluations of anti-VEGF biosimilars in retinal diseases.

**Results:**

Regulatory frameworks demonstrate rigorous biosimilarity assessment through comprehensive analytical characterization, preclinical evaluation, and clinical trials. Pivotal Phase III studies confirm therapeutic equivalence of approved biosimilars (including ranibizumab-nuna and aflibercept-abzv) with comparable visual acuity outcomes, safety profiles, and immunogenicity to originator products. Real-world evidence for ranibizumab biosimilars supports comparable outcomes to originators, while evidence for aflibercept biosimilars remains preliminary and warrants continued pharmacovigilance. Pharmacoeconomic analyses demonstrate 20–40% cost reduction compared to originators, offering substantial healthcare savings and improved patient access. Key implementation considerations include extrapolation principles, switching protocols, and clinic workflow integration.

**Conclusions:**

Although real-world data for aflibercept biosimilars are currently limited, emerging evidence for ranibizumab biosimilars supports comparable outcomes. Approved anti-VEGF biosimilars represent safe, effective, and cost-saving alternatives for retinal diseases. With robust analytical and clinical evidence supporting biosimilarity, vitreoretinal specialists can confidently adopt these agents. Successful integration requires understanding of regulatory science, evidence-based switching protocols, and healthcare system collaborations to maximize patient benefits while maintaining treatment standards. Future research should focus on long-term outcomes, broader implementation studies, and pharmacovigilance monitoring.

## Introduction

Retinal vascular diseases (RVDs) and choroidal vascular diseases (CVDs), including neovascular age-related macular degeneration (nAMD), diabetic macular edema (DME), and retinal vein occlusion (RVO) are major causes of visual impairment worldwide. Epidemiologically, the global prevalence of AMD reached approximately 196 million cases in 2020, with roughly 10% of these AMD patients progressing to develop neovascular complications (i.e., nAMD) [1, 2]. Diabetic retinopathy (DR), a microvascular complication of diabetes mellitus, affected 93 million individuals globally in 2012; among these DR patients, proliferative diabetic retinopathy (PDR) and DME accounted for 17 million and 21 million cases, respectively [3].

Over the past few decades, biotherapeutic strategies—particularly anti-vascular endothelial growth factor (anti-VEGF) agents—have revolutionized the management of retinal disease [4, 5]. Diseases previously considered irreversible causes of blindness, such as nAMD, DME, and RVO, now yield to intravitreal anti-VEGF injections, which enable significant visual improvement or even recovery, thereby substantially enhancing patients’ quality of life [5]. Therapeutic agents including ranibizumab and aflibercept, which selectively inhibit VEGF-A, effectively suppress pathological angiogenesis and vascular leakage, establishing these drugs as the standard of care for such conditions. Their clinical efficacy has been extensively validated through numerous clinical trials and real-world evidence. While biological agents have demonstrated remarkable success in ophthalmic therapeutics, their high development and manufacturing costs result in substantially higher pricing compared to conventional small-molecule drugs [5–7]. The necessity for prolonged and frequent intravitreal injections not only imposes significant financial burdens on patients but also creates substantial pressure on global healthcare systems [5, 8, 9]. This cost barrier directly compromises treatment accessibility, particularly in resource-limited settings where many patients are either unable to initiate or compelled to discontinue therapy due to financial constraints, ultimately leading to disease progression and compromised visual outcomes [9]. Consequently, the identification of more cost-effective alternatives that maintain comparable efficacy and safety profiles has emerged as a pressing challenge in ophthalmology.

With the impending expiration of patent protections for originator anti-VEGF agents such as ranibizumab and aflibercept in the United States and European markets, unprecedented opportunities have emerged for the development and commercialization of biosimilar products [4, 7, 10]. The termination of patent exclusivity disrupts the market monopoly held by originators, fostering competition that is anticipated to substantially reduce pharmaceutical costs while expanding therapeutic accessibility. This paradigm shift may enhance treatment availability for a broader patient population, facilitating timely and sustained interventions that could ultimately improve public health outcomes.

Biosimilars are biological medicinal products that demonstrate high similarity to an already licensed reference biologic product in terms of quality attributes, biological activity, safety, efficacy, and immunogenicity, with no clinically meaningful differences [11]. Unlike small-molecule generic drugs, biosimilars cannot be identical due to their structural complexity, but must demonstrate high similarity through rigorous “totality of evidence” assessments encompassing comprehensive physicochemical characterization, in vitro functional studies, pharmacokinetic (PK)/pharmacodynamic (PD) evaluations, and phase III equivalence clinical trials [7, 12]. In ophthalmology, biosimilars hold substantial therapeutic potential. They offer significant cost reductions, enhance accessibility to anti-VEGF therapies, foster market competition, generate healthcare system savings, and expand treatment options for clinicians and patients, thereby facilitating broader, more equitable, and sustainable management of ocular diseases [7, 13].

Given the escalating significance of biosimilars in ophthalmology, this review endeavors to provide retinal specialists with comprehensive evidence-based insights by comparing the efficacy and safety profiles of currently available biosimilars with their reference biologic products in ophthalmic applications. As biosimilars become increasingly prevalent, clinicians require thorough understanding of their regulatory framework, clinical data, pharmacoeconomic advantages, and practical considerations to confidently incorporate these agents into clinical practice and engage in informed discussions with patients.

## Regulatory framework and approval pathways for biosimilars

The regulatory framework and approval process for biosimilars are pivotal in ensuring their quality, safety, and efficacy demonstrate high similarity to the reference biologic product. Given the inherent complexity of biological medicines, their approval pathways differ substantially from those of small-molecule generic drugs, necessitating more rigorous and comprehensive comparative assessments [13, 14]. Major regulatory authorities worldwide, including the US Food and Drug Administration (FDA), European Medicines Agency (EMA), and World Health Organization (WHO), have established dedicated biosimilar approval pathways and continue to refine their respective guidance documents [15].

The regulatory approval of biosimilars is fundamentally based on the “totality of evidence” approach, which necessitates comprehensive comparative studies demonstrating high similarity, though not identicality, between the biosimilar and originator in terms of quality, non-clinical, and clinical attributes. This demonstration of “high similarity” follows a phased, incremental “stepwise development” process [16].

The critical components of the approval framework include:

1. Analytical and Functional Characterization: This foundational and most pivotal stage employs advanced analytical techniques (e.g., mass spectrometry, chromatography) for side-by-side comparison with the originator, establishing structural, purity, and post-translational modification (e.g., glycosylation) similarities in critical quality attributes to ensure comparable biological functionality [13, 15, 17, 18].
2. PK and PD studies: These investigations compare absorption, distribution, metabolism, excretion (PK) profiles and biological effects (PD) between biosimilar and originators. Comparable PK profiles typically predict similar drug exposure and clinical effects [13, 19].
3. Phase III equivalence clinical trials: Conducted in patient populations most sensitive to detecting potential differences, these trials demonstrate clinical efficacy and safety equivalence. For ophthalmic anti-VEGF biosimilars, best-corrected visual acuity (BCVA) and central retinal thickness (CST/CMT) typically serve as primary efficacy endpoints [7, 20, 21].
4. Immunogenicity assessment: This involves comparative evaluation of anti-drug antibody (ADA) incidence, titers, and neutralizing antibody detection to ensure the biosimilar does not elicit differential immune responses that could affect efficacy or safety [21].
5. Indication extrapolation: Based on demonstrated comprehensive similarity and understanding of disease mechanisms, regulatory authorities may permit extrapolation of efficacy and safety data to additional indications of the originator not directly studied in clinical trials, thereby reducing development costs and timelines without compromising risk–benefit understanding [22].
6. Interchangeability designation: In the US, the FDA imposes more stringent requirements for “interchangeable biosimilars,” permitting pharmacist-level substitution without physician intervention, which necessitates additional switching studies. The EMA does not establish this designation, leaving interchangeability policies to individual member states [23, 24].

While regulatory frameworks continue to improve, the approval of biosimilars still faces challenges, including variations in regulatory requirements across different jurisdictions and insufficient public awareness [24]. Future directions involve further streamlining approval requirements, enhancing international harmonization, leveraging emerging technologies (e.g., AI-based comparability modeling), and improving education and communication to facilitate broader acceptance and utilization of biosimilars.

## An Overview of approved biosimilars in Ophthalmology

**Fig. 1:**
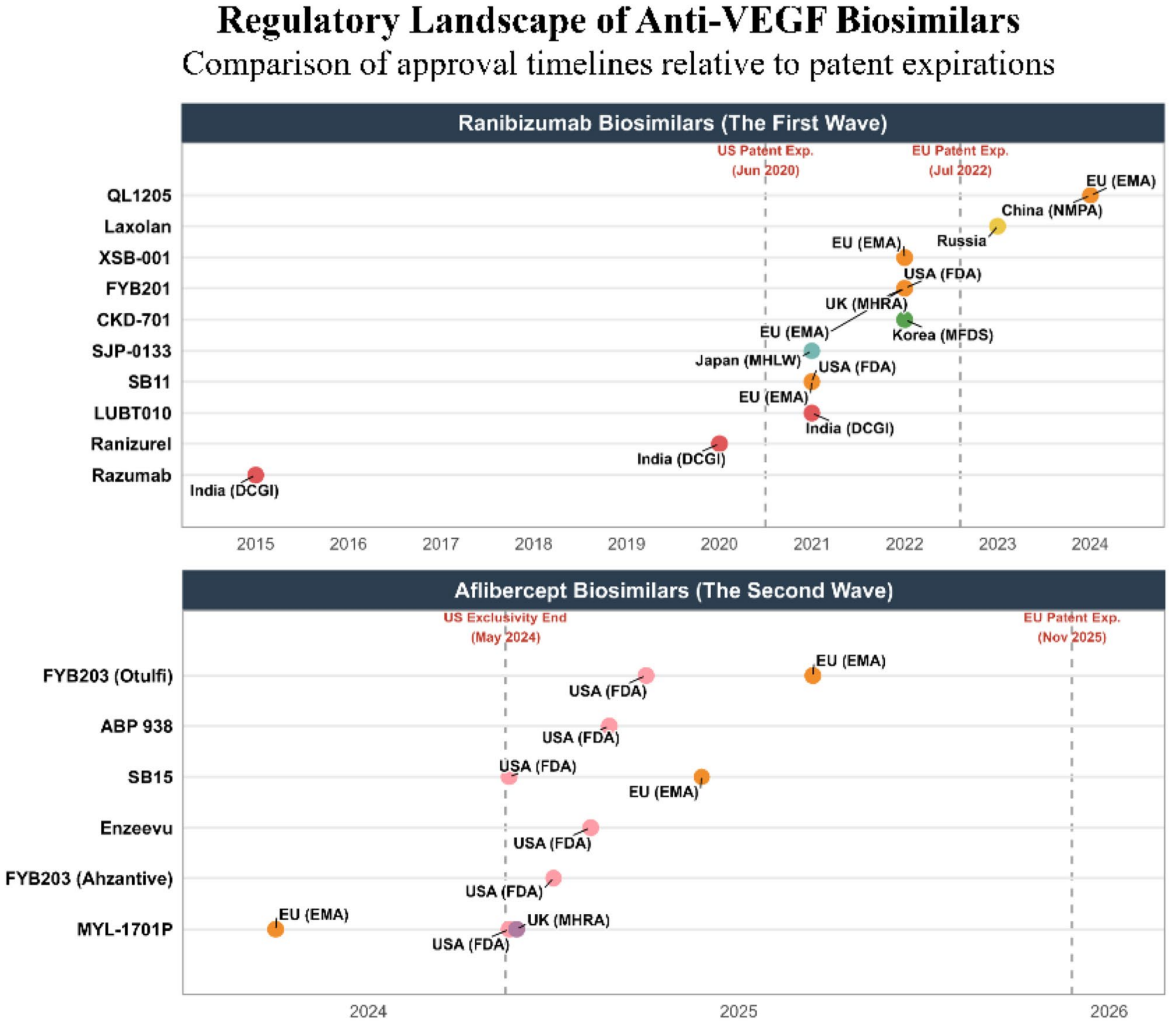
Regulatory approval timeline of anti-VEGF biosimilars relative to patent expirations. The landscape is divided into two chronological waves: ranibizumab biosimilars (top panel) and aflibercept biosimilars (bottom panel). The Y-axis lists biosimilars by their development codes or commercial names. Colored dots represent regulatory approval events across different regions (e.g., FDA, EMA, NMPA). Vertical dashed lines indicate key patent expiration or exclusivity end dates in the US and EU

Following the expiration of patents for originator anti-VEGF agents, the ophthalmic field has witnessed rapid development of biosimilar products. Current research primarily focuses on biosimilars of ranibizumab and aflibercept, two widely utilized anti-VEGF therapeutics. The regulatory approval of these biosimilars heralds a new era in ophthalmic treatment, potentially enhancing treatment accessibility for patients and healthcare system sustainability [7, 25]. The regulatory landscape and approval timelines of the first (ranibizumab) and second (aflibercept) waves of biosimilars are summarized in Fig. 1.

### Ranibizumab Biosimilars

Ranibizumab, initially developed by Genentech (USA) under the trade name Lucentis®, functions by specifically binding to VEGF-A and inhibiting its interaction with receptors. This therapeutic agent emerged as one of the first-generation anti-VEGF drugs that achieved extensive clinical utilization in ophthalmology. This therapeutic agent is indicated for the treatment of various retinal disorders, including nAMD, DME, macular edema secondary to RVO, and myopic choroidal neovascularization (mCNV). The patent for ranibizumab expired in June 2020 (US) and July 2022 (EU), precipitating a surge in biosimilar applications and approvals worldwide [26]. Currently, multiple ranibizumab biosimilars have received regulatory approval across different markets (with certain products marketed under distinct brand names in various regions). Table 1 presents detailed information regarding the currently approved ranibizumab biosimilars.

**Table 1:** Overview of major approved Ranibizumab Biosimilars

Product Name	Commercial name (country)	Developer/Collaborative Developer (Country)	Approval Authority & Approval Year	Main Indications
Razumab	-	Intas Pharmaceuticals Ltd (India)	DCGI, 2015 [27]	Reference ranibizumab
R-TPR-024	Ranizurel	Reliance Life Sciences Pvt Ltd (India)	DCGI, 2020 [28]	nAMD
LUBT010	Ranieyes	Lupin Ltd (India)	DCGI, 2021 [28]	nAMD,
			DCGI, 2022 [28]	DME, RVO, mCNV
FYB201	Ongavia (UK)	Bioeq AG (Switzerland)/Teva Pharmaceuticals (Israel)	MHRA, 2022 [29]	Reference ranibizumab
	CIMERLI (USA)	Coherus Biosciences, (USA)	FDA, 2022 [30]	
	Ranivisio (EU)	Bioeq AG (Switzerland)/Polpharma Biologic (Poland)	EMA, 2022 [31]	
XSB-001	Ximluci	STADA Arzneimittel AG (Germany)	EMA, 2022	Reference ranibizumab
Ranibizumab-nuna	Byooviz	Samsung Bioepis Co., Ltd. (South Korea)/Biogen (United States)	FDA, 2021 [32]	nAMD, macular edema following RVO, mCNV
			EMA, 2021 [33]	Reference ranibizumab
CKD-701	-	Chong Kun Dang Pharmaceutical Corporation (South Korea)	MFDS, 2022 [34]	nAMD
SJP-0133	Ranibizumab BS1	Senju Pharmaceuticals (Japan)	MHLW, 2021 [35]	nAMD, mCNV, DME
Laxolan	-	AO GENERIUM (Russia)	Minzdrav, 2023 [36]	Reference ranibizumab
QL1205	-	Qilu Pharmaceutical Co. (China)	NMPA, 2024 [37]	Reference ranibizumab
			EMA, 2024 [38]	nAMD, DME, PDR

“Reference ranibizumab” indicates approved indications extrapolated from the reference product (nAMD, DME, macular edema secondary to RVO, mCNV)

Abbreviation: DCGI: Drug Controller General of India, FDA: Food and Drug Administration (United States), EMA: European Medicines Agency, UKMHRA: UK Medicines and Healthcare products Regulatory Agency, MFDS: Ministry of Food and Drug Safety (South Korea), MHLW: Ministry of Health, Labour and Welfare (Japan), Minzdrav: Ministry of Health of the Russian Federation (Russia), NMPA: National Medical Products Administration (China), nAMD: Neovascular Age-Related Macular Degeneration, DME: Diabetic Macular Edema, RVO: Retinal Vein Occlusion, mCNV: Myopic Choroidal Neovascularization, PCV: Polypoidal Choroidal Vasculopathy, PDR:Proliferative Diabetic Retinopathy

Razumab (Intas Pharmaceuticals, Ahmedabad, India) from India represent the world’s first ranibizumab biosimilar (approved in 2015 by the Drugs Controller General of India (DCGI) prior to patent expiration) [39]. Multiple real-world studies have demonstrated comparable efficacy and safety profiles between Razumab and the originator in treating nAMD [39–41], DME [42], and RVO [43]. Subsequently, the DCGI granted approval to two ranibizumab biosimilars: R-TPR-024 (Ranizurel,Reliance Lifesciences, Mumbai, India) in 2020 and LUBT010 (Ranieyes,Lupin Limited, Mumbai, India) in 2021 [39, 44].

The first ranibizumab biosimilar approved by EMA and the FDA was SB11 (Samsung Bioepis, South Korea). This approval was supported by the RAINBOW phase III clinical trial, which demonstrated therapeutic equivalence between SB11 and the reference ranibizumab product in patients with nAMD regarding efficacy, safety, and immunogenicity profiles [20, 45]. In 2022, FYB-201 (developed by Bioeq AG) demonstrated comparable efficacy, safety, and immunogenicity to the reference ranibizumab product in the COLUMBUS-AMD phase III study. Consequently, it received approval from both the FDA and the EMA for all ranibizumab indications [26, 41]. The product was subsequently marketed under different trade names in various regions: Ongavia in the United Kingdom, CIMERLI (Ranibizumab-eqrn) in the United States, and Ranivisio® in the European Union [26]. Of note, CIMERLI received the first FDA interchangeable designation for a ranibizumab biosimilar in August 2022.

In addition, countries such as China, South Korea and Japan have also approved the marketing of ranibizumab biosimilars. The launch of these biosimilars provides more options for the treatment of retinal diseases and is expected to reduce treatment costs through market competition.

### Aflibercept biosimilars

Aflibercept, originally developed as Eylea® by Regeneron Pharmaceuticals in the United States with commercialization in European markets managed by Bayer AG, represents another widely utilized anti-VEGF therapeutic agent. Unlike ranibizumab, its mechanism of action involves the formation of a “decoy receptor” that binds both VEGF-A and placental growth factor (PlGF). This unique pharmacological property confers an extended duration of action, consequently requiring less frequent intravitreal administration compared to ranibizumab. Its patent protection in the United States concluded in May 2024. In fact, almost simultaneously with the expiration, two interchangeable biosimilars of Aflibercept - Yesafili (developed collaboratively by Mylan, Momenta Pharmaceuticals, and Biocon Biologics) and Opuviz (manufactured by Samsung Bioepis) - received FDA approval [46]. Rapidly, multiple aflibercept biosimilars have received clinical use approvals from the US FDA (6 products) and the EMA (7 products). We have compiled information regarding these approved aflibercept biosimilars in Table 2. Furthermore, numerous aflibercept biosimilars are either undergoing or have successfully completed Phase III clinical trials [62]. Their development similarly adheres to the stringent regulatory framework comparable to that of ranibizumab biosimilars. Table 2 presents detailed information regarding the currently approved Aflibercept biosimilars.

**Table 2:** Overview of major approved aflibercept biosimilars

Product Name	Commercial Name (Country)	Developer/Collaborator (Country)	Approval Authority & Approval Year	Main Indications
MYL-1701P (Aflibercept-jbvf)	Yesafili (USA, EU)	Biocon Biologies (India)	EMA (2023.09) [47]; U.S. FDA (2024.05) [46]	Reference Eylea
SB15 (Aflibercept-yszy)	Opuviz (USA, EU); Afilivu (ROK)	Samsung Bioepis (ROK)	MFDS, 2024 [48]; FDA, 2024 [46]; EMA, 2024 [49]	Reference Eylea
FYB203 (Aflibercept-mrbb)	Ahzantive (USA); Baiama (EU)	Formycon AG, Klinge Biopharma GmbH (Germany)	FDA, 2024 [50]; EMA, 2025 [51]	Reference Eylea
ABP-938 (Aflibercept-ayyh)	Pavblu (USA, EU)	Amgen (USA)	FDA, 2024 [52]; EMA, 2025 [53]	Reference Eylea
SOK583A19 (Aflibercept-shzv)	Enzeevu (USA); Afqlir (EU)	Sandoz (Switzerland)	FDA, 2024 [54]; EMA, 2024 [55]	Reference Eylea
CT-P42	Eydenzelt	Celltrion (ROK)	MFDS, 2024 [56]; EMA, 2025 [57]; FDA 2025 [58]	Reference Eylea
QL1207	-	Qilu Pharmaceutical Co. (China)	NMPA, 2023 [59]	Reference Eylea
ALT-L9	-	Alteogen (ROK)	MFDS, 2024 [60]; EMA 2025 [61]	Reference Eylea

“Reference Eylea” indicates approved indications extrapolated from the reference product (nAMD, DME, macular edema secondary to RVO, mCNV)

Abbreviation: DCGI: Drug Controller General of India, FDA: Food and Drug Administration (United States), EMA: European Medicines Agency, UKMHRA: UK Medicines and Healthcare products Regulatory Agency, MFDS: Ministry of Food and Drug Safety (South Korea), NMPA: National Medical Products Administration (China)

### Bevacizumab: off-label use and biosimilar challenges

Bevacizumab (originally developed by Genentech under the brand name Avastin®), which exhibits broad-spectrum binding affinity for VEGF family isoforms, has demonstrated clinical utility beyond its initial oncological indications as an anti-VEGF monoclonal antibody. Its off-label application for retinal disorders has gained widespread adoption across multiple jurisdictions, primarily attributable to its favorable pharmacoeconomic characteristics [4, 63]. Off-label repackaged bevacizumab (priced at $90 per dose under Medicare reimbursement in the United States) currently represents the sole cost-containment strategy for anti-VEGF therapy. The CATT trial in 2011 confirmed that bevacizumab was non-inferior to ranibizumab in the treatment of wet AMD [64]. In the United States, off-label repackaged bevacizumab has been reported to cost approximately $50–$90 per dose under Medicare reimbursement, compared with approximately $2,000 for ranibizumab—a price ratio of roughly 1:20 to 1:40 [65]. Several approved and investigational bevacizumab biosimilars exist, including ABP215 (Mvasi) developed by Amgen (Thousand Oaks, CA, USA) and Allergan (Dublin, Ireland), which received FDA and EMA approval in 2017 [66]. However, due to bevacizumab’s global economic accessibility and ophthalmologists’ established confidence in its use, these biosimilars are primarily employed in oncology rather than ophthalmology. Regulatory attitudes toward ophthalmic-specific bevacizumab diverge between the FDA and EMA. The EMA approved LYTENAVA (bevacizumab gamma, previously ONS-5010) in May 2024 based on demonstrated biosimilarity to reference bevacizumab for the treatment of wet AMD, addressing off-label repackaging risks [67]. Notably, in the EU, LYTENAVA was approved through a biosimilar pathway. In the United States, however, the FDA has rejected the corresponding applications due to Chemistry, Manufacturing, and Controls (CMC) concerns, and ONS-5010 was developed under a BLA rather than a biosimilar pathway. To date, only limited clinical studies have been published regarding intravitreal injection of bevacizumab biosimilars, predominantly from low- and middle-income countries [68–70].

## Comparative analysis of efficacy and safety

The key aspect of biosimilars lies in demonstrating their high similarity or equivalence in clinical efficacy compared to the reference originator product. This is typically achieved through rigorously designed phase III randomized controlled clinical trials conducted in patient populations, which are most sensitive for detecting potential differences. For ophthalmic anti-VEGF biosimilars, the primary efficacy endpoints of interest include improvement in BCVA and reduction in central subfield thickness (CST/CMT) [44].

### Analysis of efficacy and safety of Ranibizumab Biosimilars

The efficacy evaluation of ranibizumab biosimilars primarily focuses on patients with neovascular nAMD, as nAMD represents one of the principal indications for anti-VEGF therapies, and disease progression along with treatment response demonstrates particular sensitivity to pharmacological variations. Multiple pivotal phase III clinical trials and real-world studies have yielded robust evidence regarding the therapeutic efficacy of ranibizumab biosimilars.

#### Evidence of equivalence between the results of key clinical trials and the original research drug

The RAINBOW study of SB11 (Byooviz) represents the pivotal phase III clinical trial for the first ranibizumab biosimilar approved by both EMA and FDA. This multicenter investigation compared the efficacy, safety, and immunogenicity of SB11 with the originator (Lucentis®) in patients with nAMD. The primary analysis demonstrated comparable efficacy, with mean BCVA improvements from baseline of 6.2 and 7.0 letters in the SB11 and reference groups at week 8, respectively, meeting the predefined equivalence margin [20]. Secondary endpoints, including reductions in CST, similarly confirmed therapeutic equivalence. The study also reported comparable safety and immunogenicity profiles. Follow-up data spanning 52 weeks demonstrated sustained equivalence in longitudinal outcomes, with no statistically significant differences observed in injection frequency requirements, safety parameters, or immunogenicity profiles [45].

The phase III trial of CKD-701 employed a pro re nata (PRN) regimen in nAMD patients to evaluate comparative efficacy versus reference ranibizumab. At the 3-month assessment, both treatment arms exhibited comparable proportions of patients losing fewer than 15 BCVA letters, with no statistically significant differences observed in mean BCVA improvement or CRT changes. CKD-701 also exhibited comparable efficacy, safety, and immunogenicity profiles to the reference ranibizumab, while successfully achieving and maintaining visual acuity improvements through the PRN regimen [71].

The clinical development program of FYB201 similarly demonstrated therapeutic equivalence in patients with wAMD. Comparable best - corrected visual acuity outcomes were observed between the biosimilar and reference groups, accompanied by statistically significant reductions in mean central macular thickness at week 24. With no clinically meaningful differences in terms of safety, efficacy, and immunogenicity profiles between FYB201 and the reference ranibizumab [72], supporting the absence of clinically meaningful differences.

The accumulated evidence from these trials demonstrates therapeutic equivalence between ranibizumab biosimilars and the originator in the treatment of nAMD. A systematic review and meta-analysis conducted by Hatamnejad et al. [73], which incorporated data from four randomized controlled trials (including FYB201, SB11, RanizuRel, and Lupin’s ranibizumab) involving 1,544 eyes, further corroborated comparable efficacy in both functional (best-corrected visual acuity, BCVA) and anatomical (central subfield thickness/central macular thickness, CST/CMT) outcomes. Notably, while significant heterogeneity was observed for intraocular pressure-related adverse events (AEs) (I^2^ = 76%), the analysis revealed no statistically significant differences in treatment-emergent AEs (risk ratio 1.06, 95% confidence interval 0.91–1.23, *p* = 0.45) or intraocular pressure-related AEs (risk ratio 2.59, 95% confidence interval 0.11–62.25, *p* = 0.56).

### Analysis of the efficacy data of aflibercept biosimilars

Although the patent expiration of aflibercept occurred later than that of ranibizumab, pharmaceutical companies have demonstrated substantial interest in developing its biosimilars. Several phase III equivalence clinical trials have been conducted to compare the efficacy and safety profiles between multiple aflibercept biosimilars and the originator product across indications including nAMD, and DME.

The phase III trial of SB15 (aflibercept-yszy) (SB15-224/AFL-225) focused on nAMD and incorporated an innovative treatment-switching design, enrolling 449 nAMD patients [74]. The study design featured initial randomization to SB15 or reference aflibercept for 32 weeks, followed by 1:1 re-randomization of the reference group to either continue reference treatment or switch to SB15. The primary endpoint evaluated BCVA improvement at week 8 [equivalence margin ±3.0 Early Treatment Diabetic Retinopathy Study (ETDRS) letters]. Results showed a between-group difference of 0.1 letters (95% CI: −1.3 to 1.4), meeting equivalence criteria. Post-switch assessments at week 40 demonstrated comparable BCVA outcomes between the switched and continuous reference groups, confirming therapeutic stability following transition from reference to biosimilar. Secondary CST outcomes showed similar reductions at week 8, with maintained anatomical equivalence through 56 weeks and no treatment-related fluctuations observed post-switch. In terms of safety, there were no clinically relevant differences in the incidence of AEs.

The phase III trial of FYB203 (aflibercept-mrbb) (FYB203-215/EU-Eylea-218) investigated nAMD patients in a 1:1 randomized, double-blind, multicenter study (*N* = 433) with a prespecified equivalence margin of ±3.5 ETDRS letters (aligned with EMA standards) [75]. Primary endpoint analysis revealed a week 8 BCVA improvement difference of 1.0 letters (90.4% CI: −0.3 to 2.2) between FYB203 and reference groups, meeting equivalence requirements. Secondary outcomes at week 4 demonstrated comparable CST reductions (−171.4 μm versus −166.9 μm), and at week 24, similar proportions of patients achieved ≥ 10 ETDRS letter gains (20.4% versus 19.2% in both groups). Safety profiles were consistent between groups (TEAE: 76.7% vs 72.5%; SAE: 7.9% vs 10.6%), with low and comparable immunogenicity (new ADA: 1.0% vs 0.5%). These findings support long-term therapeutic equivalence and safety of aflibercept biosimilars in nAMD, with the study’s sample size providing 90% statistical power for robust conclusions.

The phase III trial of ABP-938 (aflibercept-ayyh) constituted a large-scale nAMD investigation (*N* = 576) employing a 16-week switching design (reference group re-randomized 1:1 at week 16 to either continue reference or switch to ABP-938) [76]. Primary analysis showed a week 8 BCVA improvement difference of −0.1 letters (90% CI: −1.1 to 1.3), satisfying the ±3.0 ETDRS letter equivalence margin. At week 40 (24 weeks post-switch), visual acuity maintenance was comparable between switched and continuous reference groups, confirming mid-term therapeutic stability following biosimilar transition. Secondary CST outcomes demonstrated > 150 μm reductions at week 8 and sustained anatomical improvements at week 52, with >90% of patients maintaining < 15 ETDRS letter loss in both groups, validating functional and anatomical equivalence. In terms of ocular safety, no clinically significant differences were observed in the incidence of ocular or non-ocular AEs between the two groups. Ocular safety profiles were comparable between groups, with no clinically meaningful differences observed in the incidence of ocular AEs or systemic AEs. Immunogenicity assessments demonstrated low and comparable rates of ADA formation and binding antibody development across all treatment groups. Furthermore, a pharmacokinetic substudy confirmed minimal and comparable systemic exposure for both ABP-938 and the originator.

The phase 3 MYLIGHT study (*N* = 484) demonstrated clinical equivalence between the investigational SDZ-AFL (SOK583A19; afqlirumab, Afqlir®) and the reference aflibercept in patients with nAMD [77, 78]. The primary endpoint at week 8 revealed a best-corrected visual acuity (BCVA) difference of −0.3 letters (90% confidence interval: −1.5 to 1.0), meeting the equivalence margins established by both the US Food and Drug Administration (±3.0 letters) and the EMA (±3.5 letters) (representing one of the few studies to concurrently satisfy both regulatory agencies’ equivalence criteria for nAMD). Comparable safety profiles and immunogenicity outcomes were observed across treatment arms.

QL1207 (Qilu Pharmaceutical Co., Ltd., Jinan, China) represents the first aflibercept biosimilar developed in China. In a multicenter phase 3 clinical trial involving 366 patients with neovascular nAMD, QL1207 demonstrated comparable efficacy, similar safety profiles, equivalent immunogenicity, analogous pharmacokinetic characteristics, and equivalent VEGF inhibitory effects when compared to the originator aflibercept [79].

Beyond nAMD, biosimilars of aflibercept have also been clinically evaluated in other approved indications such as DME through cohort studies.

The phase III INSIGHT clinical trial evaluated the comparative efficacy and safety of the aflibercept biosimilar MYL-1701P (aflibercept-jbvf) versus the reference aflibercept in patients with DME [80]. This randomized study enrolled 355 participants, with 179 allocated to the MYL-1701P arm and 176 to the reference aflibercept arm. The primary endpoint analysis at week 8 demonstrated comparable improvements in best-corrected visual acuity (BCVA), with mean changes of 6.60 ± 0.55 letters in the MYL-1701P group versus 6.56 ± 0.55 letters in the reference group (adjusted mean difference: 0.04 letters; 90% CI: −1.16 to 1.24), meeting the predefined equivalence criteria. Anatomical outcomes revealed central subfield thickness (CST) reductions of −112±7 μm and −124±7 μm in the respective treatment groups at week 8. The safety profile was similar between arms, with comparable rates of ocular AEs (30.9% vs 29.5%) and low incidence of serious ocular AEs (0.6% vs 1.1%). These findings substantiate the therapeutic equivalence of MYL-1701P to the reference aflibercept in the management of DME.

The phase III clinical trial of CT-P42 (CT-P42-173/AFL-175) evaluated the efficacy and safety of CT-P42 compared to reference aflibercept in 306 patients with DME. This randomized, double-masked study employed a 1:1 allocation ratio, with the primary endpoint being the mean change in BCVA from baseline at week 8. At week 52, the BCVA improvement was 12.1 letters in the CT-P42 group versus 11.1 letters in the reference aflibercept group [81]. The mean change in central subfield thickness was −176.8 μm for CT-P42 and −172.4 μm for the originator. The study demonstrated comparable efficacy, safety, and immunogenicity profiles between the two treatment arms.

## Comparative analysis of real-world evidence Data

While randomized clinical trials provide high-level evidence, RWE plays a crucial role in evaluating the performance and long-term safety profiles of biosimilars across broader, more heterogeneous patient populations.

### Real-world evidence for ranibizumab biosimilars

As the first biosimilar of ranibizumab approved for ophthalmic use, Razumab has been extensively studied in real-world settings. Multiple Indian studies have assessed the therapeutic outcomes of Razumab in various retinal disorders. Sharma et al. demonstrated in their prospective ASSET study that the biosimilar ranibizumab (Razumab™) showed comparable safety and efficacy to the originator in wet AMD patients, with significant improvements in visual acuity and no new safety signals identified [82].

Ratra et al. reported in their retrospective comparative study that biosimilar ranibizumab (type not specified) demonstrated comparable efficacy and safety to both innovator ranibizumab and bevacizumab in the treatment of nAMD and macular edema, with no significant differences observed among the three anti-VEGF agents at any time point during the 3–24 month follow [83].

A retrospective multicenter study compared the efficacy and safety of Razumab versus ranibizumab for the treatment of myopic choroidal neovascular membranes [84]. The biosimilar ranibizumab (Razumab®) demonstrated non-inferior efficacy and safety profiles when compared to the reference ranibizumab. During the 12-month follow-up period, both agents exhibited comparable outcomes in terms of visual acuity improvement, reduction in central macular thickness, and injection frequency.

A systematic review by Chatzimichail et al. (2024) further corroborated that ranibizumab biosimilars exhibit comparable efficacy and safety to originators in treating various retinal disorders including nAMD, ROP, DME, and polypoidal choroidal vasculopathy (PCV), while offering cost advantages in resource-limited settings (Chatzimichail et al., 2024). These RWE findings collectively reinforce the clinical reliability of ranibizumab biosimilars.

### Real-world evidence for aflibercept biosimilars

Due to their relatively recent market entry, published real-world evidence for aflibercept biosimilars remains quite limited.

A retrospective, uncontrolled observational study conducted in Iran evaluated the early real-world safety and efficacy outcomes following administration of aflibercept 2 mg biosimilar (Tyalia, manufactured by Cinnagen, Tehran, Iran). The analysis included 102 eyes with n-AMD, 67 eyes with DME, and 20 eyes with RVO-associated macular edema. Results demonstrated a statistically significant improvement in mean central subfield thickness (CST) from baseline (408.8±155.1 μm) to final follow-up (353.4±142.4 μm; *p* < 0.001) across all treatment groups. Best-corrected visual acuity remained stable throughout the study cohort. The overall AE rate was 0.4%. These preliminary real-world findings from this limited early series suggest comparable clinical efficacy and safety profiles between Tyalia and the reference aflibercept 2 mg product within its approved indications.

### Safety assessment of switching studies

Interchangeability refers to the clinical substitution of a biosimilar for its originator without requiring physician intervention, while maintaining comparable efficacy and safety profiles without increased risks or diminished therapeutic effects [85]. The FDA mandates specific requirements for demonstrating interchangeability, typically necessitating additional switching studies [86]. In contrast, regulatory guidelines from the EMA and World Health Organization (WHO) consider biosimilars interchangeable upon approval without requiring supplementary switching studies [87].

In current clinical experience with antitumor therapy, switching from originator biologics to biosimilars has demonstrated no significant differences in immunogenicity, safety, or efficacy profiles. The observed safety signals remain consistent with those of the originators [88, 89]. These positive switching experiences provide important reference for the safe transition to anti-VEGF biosimilars.

Multiple clinical studies on anti-VEGF biosimilars have specifically designed switching protocols to evaluate safety issues during the transition process. The phase III trial of SB15 in nAMD patients incorporated a “32-week switching regimen” design, with safety monitoring encompassing three cohorts: “continuous biosimilar,” “continuous originator,” and “originator-to-biosimilar switch” [74] Results demonstrated comparable ADA positivity rates across groups (2.1%, 2.3%, and 1.9%, respectively; *p* > 0.05). Regarding ocular AEs (e.g., vitreous opacities, transient intraocular pressure elevation), they occurred in 25%-27% of patients across cohorts, while systemic AEs (e.g., headache, hypertension) maintained incidence rates below 10%. No new safety signals emerged post-switch (including abrupt ADA titer elevation or emergence of unique AEs). Extended 56-week follow-up data corroborated the comparable safety profiles between SB15 and the originator, irrespective of treatment sequence, with no evidence of dose-dependent or time-dependent safety variations.

In the phase III randomized trial (ABP-938–288/AFL-288) for nAMD patients, a 16-week treatment-switching design was employed to evaluate immunogenicity and safety before and after transitioning [76]. Results showed that the incidence of binding anti-drug antibodies (ADAs) was comparable across the continuous ABP-938 group (2.5%), switch-to-ABP-938 group (2.3%), and continuous originator group (2.7%). Treatment-emergent AEs (TEAEs) remained stable (26–28%) across all groups 4 weeks post-switch (i.e., at week 20), with no switching-related peak in AEs such as increased ocular irritation. Throughout the 52-week follow-up, the incidence of neutralizing antibodies in the ABP-938 group remained below 0.5%, consistent with the originator group, and no long-term safety concerns were identified.

A small-scale Indian study retrospectively analyzed 30 eyes with various retinal vascular disorders (13 eyes with DME, 5 with branch RVO, 2 with central RVO, and 10 with nAMD) in 20 patients who were switched from the reference ranibizumab to Razumab in a consecutive case series [90]. During the 6-month follow-up period, no clinical immunogenicity concerns were observed, and therapeutic efficacy was maintained. Ninety percent of patients continued biosimilar treatment, with visual acuity outcomes remaining stable or demonstrating improvement.

Collectively, current clinical research data indicate that switching to anti-VEGF biosimilars is reliable from a safety perspective. Neither immunogenicity indicators nor AE incidence rates showed significant differences between switching groups and continuous treatment groups. These results support the safe substitution of anti-VEGF biosimilars for originator products in clinical practice without additional safety risks or immunogenicity concerns. This finding has important implications for expanding patient access to high-quality, economically affordable anti-VEGF treatments.

## Impact of formulation and device characteristics on clinical safety and usability

While regulatory scrutiny is rigorously applied to the structural and functional similarity of the active biological molecule, the drug product formulation (excipients) and drug delivery system (primary container closure system) often exhibit significant variations. These ‘non-molecular’ discrepancies represent a critical, yet frequently underestimated, determinant of clinical safety and adoption in ophthalmology.

Biosimilar developers frequently modify buffer systems or stabilizers to circumvent originator patents or enhance stability. However, alterations in excipients (e.g., transitioning from histidine-based to phosphate-based buffers) may subtly influence protein unfolding or aggregation rates under stress conditions [91]. In the context of intravitreal administration, protein aggregates serve as potent drivers of immunogenicity, potentially precipitating ADAs or sterile IOI [92]. Although current biosimilars have demonstrated comparable safety profiles in Phase III trials, these studies are typically powered to detect common AEs rather than rare, formulation-driven inflammatory signals, necessitating rigorous post-marketing surveillance.

Furthermore, pivotal Phase III equivalence trials primarily prioritize visual acuity outcomes, whereas’device usability’—specifically regarding potential variations in the mechanical performance of prefilled syringes (PFS) is often relegated to secondary or exploratory endpoints. Consequently, there is a paucity of comparative data regarding the’physician experience,‘particularly concerning syringe malfunction rates, needle sharpness, or subtle injection-related pain scores [93]. As the market becomes saturated with multiple biosimilar options, differentiation may shift from‘molecular efficacy’ (which is assumed equivalent) to’device reliability.‘Future real-world studies must specifically capture device-related AEs (e.g., syringe jamming, needle detachment) to reassure the ophthalmic community that cost savings are not achieved at the expense of procedural safety

## Pharmacoeconomic Analysis of Biosimilars

One of the most notable advantages of biosimilars lies in their pharmacoeconomic value, which manifests through the provision of more cost-effective treatment options, reduction in healthcare expenditures, and improved patient access to therapies. This holds profound significance for high-cost biologics, particularly in ophthalmology where long-term, frequent injections are often required.

### Cost savings and market competition

The introduction of biosimilars aims to break the market monopoly of originator biologics and reduce drug prices through competition. Numerous studies have confirmed that biosimilar market entry indeed generates substantial cost-saving effects. By enhancing the accessibility of biologic therapies, biosimilars have reduced treatment waiting times and facilitated the introduction of innovative treatments [94]. Chen et al. (2024) examined annual sales data of eight biological products across 57 countries and regions from January 1, 2012, to December 31, 2019. Their findings demonstrate that the introduction of biosimilars has significantly reduced the prices of biologics both globally and in the United States, with continued annual price reductions observed [95]. Europe has demonstrated leadership in the adoption of biosimilars, with the first biosimilar monoclonal antibody Remsima (Inflectra) receiving approval in the EU market as early as 2013. This initiative has yielded substantial cost savings: estimates indicate savings of approximately $11.8 billion during the period from 2016 to 2022 alone [25]. Gokul S and colleagues recently assessed the biosimilar revolution in the European Union and reported that the comprehensive regulatory structure has enhanced accessibility and reduced healthcare costs, though challenges in stakeholder acceptance and market barriers persist [96]. In the U.S. market, despite the growing number of biosimilar approvals, their market penetration and actual cost savings continue to face challenges, including payer preference for rebates which may impede market share expansion [97]. However, in the oncology sector, the introduction of trastuzumab biosimilars has demonstrated significant reductions in healthcare expenditures without compromising recurrence rates, achieving a market share increase from 7% to 32% within two years [98].

### Economic burden of retinal diseases and cost-saving potential of Biosimilars

Retinal diseases impose substantial economic burdens on healthcare systems worldwide.Taking nAMD as an illustrative case, Almony et al. (2021) quantified the clinical and economic burden of nAMD in a large U.S. commercially insured cohort and found that patients with active CNV incurred mean nAMD-related outpatient costs of $8,658 versus $2,406 for inactive CNV and $1,198 for inactive scar (≈4- and 7-fold higher, respectively; *p* < 0.0001) [99]. Anti-VEGF injection frequency and drug choice were the dominant cost drivers: ≥10 injections increased costs by 342% relative to 1–3 injections, and initial therapy with ranibizumab or aflibercept raised costs by 268–272% compared with bevacizumab (all *p* < 0.0001) [99].

European studies have similarly highlighted the cost implications of nAMD treatment in relation to anti-VEGF therapy management: A 2021 quantification analysis of hospital-level direct costs for nAMD in Spain revealed that, under routine care, mean annual expenditure per patient reached €4,628 (total €5.39 million across 1,164 patients in five tertiary hospitals over 1 year), with optical-coherence-tomography examinations (€2.83 million) and anti-VEGF therapy (€2.04 million) accounting for the largest shares; extrapolation to the national population ≥ 65 y suggested nAMD consumes €0.98–1.46 billion (1.4–2.1% of total public health spending) [100]. Overall, in 2023, according to industry market research reports, the global market size for AMD-related treatments exceeded $10 billion, with projections indicating it will approach $18 billion by 2030 [101]. Concurrently, according to industry market research reports, the DME market reached $5.4 billion [102], while the PDR market is projected to exceed $2.7 billion by 2025 [103].

An analysis examining national and Rhode Island-specific utilization patterns of VEGF inhibitors utilizing Medicare Part B and Rhode Island All-Payer Claims Database (RI APCD) data revealed that these therapeutic agents accounted for substantial expenditures within the Medicare Part B program, exceeding $4.5 billion in 2022 alone [104].

The market entry of biosimilars enhances patient access to treatment through price reduction, thereby mitigating global disparities in biologic medicine availability. A Spanish study analyzed patients with immune-mediated inflammatory diseases initiating biologic therapy with either originator drugs or biosimilars (infliximab, etanercept, adalimumab) [105]. The biosimilars significantly enhanced accessibility to biologic treatments, reducing the waiting time for therapy by 1.6 years. In the field of ophthalmology, the introduction of ranibizumab and aflibercept biosimilars is anticipated to yield substantial pharmacoeconomic benefits. Budget impact modeling demonstrated that broader adoption of biosimilars priced at 65% of originator costs could yield substantial annual savings for aflibercept and ranibizumab, ranging from $455 million to $1.06 billion in the US market [104].

Yanagi et al. constructed a 20-year Markov cohort model with 3-month cycles, incorporating Japanese visual acuity, treatment frequency, and utility data to evaluate lifetime costs and quality-adjusted life years (QALYs) [106]. The study compared ranibizumab biosimilar versus aflibercept across three nAMD subtypes from a societal perspective, and versus branded ranibizumab, aflibercept, aflibercept-to-biosimilar switching, and best supportive care from the patient perspective (with explicit modeling of age-specific co-payment rules and the national high-cost medical cap). Their analysis demonstrated that ranibizumab biosimilar yielded lifetime savings of JPY 50,447–1,286,570 (≈US$330–8,450) per patient compared to aflibercept across nAMD subtypes. Additionally, it reduced patient co-payments by JPY 81,469–391,935 (≈US$535–2,575) (with co-payment cap) or JPY 138,948–391,935 (≈US$910–2,575) (without cap) relative to branded ranibizumab or aflibercept treat-and-extend regimens. Another study from the same research group also demonstrated that for treatment-naïve nAMD patients in Japan, both the treat-and-extend regimen and PRN approach with ranibizumab biosimilars resulted in cost savings while generating comparable or slightly greater QALYs when compared to originator ranibizumab, aflibercept, or best supportive care [8].

India, as the first country globally to introduce a biosimilar version of ranibizumab, has provided valuable insights for healthcare systems worldwide through its pioneering experience. The introduction of biosimilars in 2015 precipitated a significant pricing transformation in the Indian pharmaceutical market. Within less than a decade, the annual treatment cost plummeted from 420,000 INR to 36,000 INR, representing a dramatic 97% reduction. This dramatic price breakthrough not only enhanced treatment accessibility but also fundamentally restructured the market landscape. With multiple biosimilar manufacturers entering the market, escalating competitive pressures compelled originator companies to implement strategic responses, including price reductions for their branded products and the introduction of patient assistance programs [107].

Different nations implement distinct reimbursement mechanisms within their respective healthcare systems to facilitate biosimilar adoption: In European markets, biosimilars typically enjoy price discounts of 20%-35% compared to their reference biologic products [108]. The U.S. market employs a unique reimbursement calculation formula for biosimilars: the average sales price (ASP) of the biosimilar plus 6% of the reference biological product’s ASP [109].

### The bevacizumab Paradox: price escalation risk in ophthalmic biosimilars

While the market entry of ranibizumab and aflibercept biosimilars follows a conventional ‘price erosion’ model—driving acquisition costs of high-priced biologics downward through competition—the potential commercialization of ophthalmic-labeled bevacizumab presents a unique ‘price escalation’ risk. Currently, off-label repackaged bevacizumab functions as the global economic ‘floor’ for anti-VEGF therapy (approx. $50–$90 per dose). However, the approval of an ophthalmic-specific bevacizumab biosimilar could trigger regulatory mechanisms (such as the Drug Quality and Security Act in the US) that prohibit the compounding of commercially available drugs, effectively eliminating the low-cost off-label option [110]. This regulatory shift would force a transition from the low-cost compounded product to a significantly higher-priced commercial biosimilar. Quantitative modeling by Zhang et al. estimates that, depending on the pricing strategy of the new ophthalmic bevacizumab, this mandatory substitution could increase Medicare Part B spending by $494 million to $7.6 billion annually [110]. This reverse economic impact could potentially neutralize or even exceed the cumulative savings generated by the adoption of ranibizumab and aflibercept biosimilars. Consequently, policymakers face a complex dilemma: the pursuit of enhanced pharmaceutical quality standards (via FDA-approved ophthalmic bevacizumab) may paradoxically compromise the financial sustainability of retinal disease management. However, for policymakers and clinical ophthalmic practice, it is also imperative to acknowledge the safety risks associated with pharmacy repackaging of off-label bevacizumab (such as endophthalmitis outbreaks) [111].

In some emerging economies, the mechanism for achieving cost-containment extends beyond passive market competition between originators and biosimilars. State-orchestrated initiatives, such as Brazil’s “Partnerships for Productive Development” (PDP), exemplify a strategic pivot from importation to localized technological autonomy. By facilitating technology transfer from multinational entities to local public laboratories (e.g., Bio-Manguinhos), these partnerships aim to secure the supply of high-cost biologics—including rituximab and bevacizumab—thereby insulating domestic healthcare systems from global pricing volatility [112, 113].

This PDP model fundamentally alters the pharmacoeconomic calculus regarding the “Bevacizumab Paradox.” Rather than facing a binary choice between unregulated off-label compounding and premium-priced biosimilars, such frameworks foster the production of high-quality, state-subsidized biologics. Samsung Bioepis, for instance, has leveraged this pathway to supply etanercept biosimilars, a precedent likely to influence the trajectory of ophthalmic anti-VEGF adoption [112]. For the retina community in the Global South, these localized production ecosystems offer a sustainable third avenue, potentially mitigating the risk of cost-prohibitive regulation while ensuring adherence to rigorous GMP standards absent in informal compounding pharmacies.

### The durability challenge: extended-interval agents in the pharmacoeconomic landscape

The cost-savings analysis of anti-VEGF biosimilars must be contextualized within a competitive landscape increasingly shaped by extended-interval therapeutic alternatives. Two agents—high-dose aflibercept 8 mg (Eylea HD) and faricimab (Vabysmo)—have demonstrated the ability to maintain or improve clinical outcomes while substantially reducing injection frequency, thereby directly reducing the number of intravitreal injections, clinic visits, imaging studies, and chair-time required per patient per year.

High-dose aflibercept 8 mg was evaluated in the PULSAR Phase III trial (*n* = 1,009), which demonstrated that approximately 83% of patients maintained every-12-week dosing and 77% maintained every-16-week dosing at 48 weeks, with non-inferior visual acuity gains compared to aflibercept 2 mg every 8 weeks [114]. Faricimab, a bispecific antibody targeting both VEGF-A and Ang-2, was investigated in the TENAYA and LUCERNE Phase III trials (*n* = 1,329) for nAMD. Approximately 45% of patients maintained every-16-week dosing through year 1, with approximately 80% maintained on every-12-week or longer intervals [115]. These durability profiles translate into an estimated 3–5 annual injections for Eylea HD and 3–7 for faricimab, compared with 6–8 injections for a 2 mg aflibercept biosimilar under an every-8-week regimen.

From a pharmacoeconomic standpoint, the reduction in injection frequency carries tangible economic implications. Even at originator pricing, the annual per-patient cost of aflibercept 8 mg or faricimab at every-12 to every-16 week intervals may approach or undercut the total cost of a 2 mg aflibercept biosimilar administered every 8 weeks, particularly when indirect costs (e.g., travel, caregiver time, lost productivity) and the cumulative burden of procedure-related expenses (e.g., injection fees, imaging) are factored into the analysis. For instance, assuming conservative Medicare reimbursement rates, a patient receiving 4 injections per year of Eylea HD (at approximately $2,000 per dose) would incur roughly $8,000 in drug costs, while a patient receiving 7 injections per year of a 2 mg aflibercept biosimilar (at approximately $1,200 per dose) would incur roughly $8,400—a difference that narrows further or reverses when the cost of 3 fewer clinic visits and imaging sessions is included.

Therefore, biosimilar developers face a strategic challenge: the cost-per-vial advantage of 2 mg aflibercept biosimilars may be partially or fully offset by the durability premium offered by longer-acting alternatives, especially in patient populations with high treatment burden or limited access to frequent clinic visits. The value proposition of biosimilars is likely strongest in health systems where per-injection costs are low and patients are closely monitored, and relatively weaker in settings where durability-driven savings on indirect costs predominate. A comprehensive pharmacoeconomic model for anti-VEGF therapy should therefore incorporate these competing dynamics rather than focusing solely on per-vial price differentials.

## Considerations in clinical practice

The introduction of biosimilars has expanded therapeutic options for retinal specialists while necessitating careful evaluation of multiple factors in clinical practice, including the scientific characteristics of the agents, clinician and patient perceptions, psychological expectations, and healthcare system management strategies.

### Clinician and patient perceptions and confidence

The successful adoption of biosimilars is contingent upon the confidence of healthcare professionals and patients in their efficacy and safety profiles. A survey by Sharma et al. evaluating retinal specialists in the United States and Europe revealed a dichotomy: while 56.3% of respondents were familiar with anti-VEGF biosimilars, approximately 50% expressed lingering concerns regarding safety, efficacy, immunogenicity, and indication extrapolation. Notably, American physicians demonstrated significantly greater apprehension regarding biosimilar safety (*p* = 0.0371) and effectiveness compared to their European counterparts, correlating with a stronger preference for continuing off-label use of bevacizumab [116]. Experience from oncology suggests that clinician hesitation often stems from concerns over interchangeability and the potential impact of multiple substitutions on clinical outcomes [117]. This hesitation can inadvertently transfer to patients. Effective physician-patient communication is critical; clinicians must clearly articulate the scientific rationale, rigorous regulatory approval processes (“totality of evidence”), and robust clinical data demonstrating biosimilarity. Failure to bridge this knowledge gap may precipitate the “nocebo effect”—a phenomenon where negative expectations regarding a therapeutic intervention lead to perceived adverse outcomes or treatment discontinuation, independent of the drug’s pharmacological properties [118, 119]. Studies on biosimilar switching indicate that higher discontinuation rates are often driven by subjective patient-reported outcome deterioration rather than objective clinical parameters [120, 121]. Therefore, proactive and evidence-based communication is paramount to establishing patient trust and mitigating nocebo-related treatment failures.

### Understanding of interchangeability and indication extrapolation

“Interchangeability” represents a distinct regulatory designation, particularly within the United States context. The FDA imposes stringent standards for “interchangeable biosimilars,” often requiring additional switching studies to demonstrate that alternating between the biosimilar and originator does not increase safety risks or diminish efficacy [86]. This designation permits pharmacist-level substitution without prescriber intervention [122]. In contrast, the EMA does not utilize a specific “interchangeable” label, leaving substitution policies to individual member states [123]. For ophthalmologists, particularly in “buy-and-bill” healthcare models, retaining control over therapeutic selection remains essential to ensure treatment continuity and accommodate patient-specific factors, regardless of automatic substitution permissions.

Closely linked to this is the concept of indication extrapolation. This regulatory principle allows a biosimilar to be approved for indications not directly studied in clinical trials, provided that high similarity is demonstrated in the most sensitive population and the mechanism of action is consistent across conditions [124]. While extrapolation is scientifically grounded in the “totality of evidence” and enhances cost-effectiveness, it remains a source of skepticism among clinicians. Sharma et al. reported that 67.8% of retinal specialists expressed reservations regarding extrapolation [26]. Bridging the gap between regulatory science and clinical practice requires continuous education to reassure practitioners that extrapolated indications are supported by robust physicochemical and functional comparability data, not merely assumption.

### Longitudinal data and real-world evidence

While pivotal clinical trials demonstrate therapeutic equivalence, they are often limited by strict inclusion criteria and relatively short durations. Post-marketing surveillance and RWE are therefore indispensable for validating the long-term safety and effectiveness of biosimilars in broader, heterogeneous patient populations (e.g., those with significant comorbidities) [14]. Ranibizumab biosimilars have already accumulated substantial RWE confirming their clinical utility. Conversely, as aflibercept biosimilars are recent market entrants, continued generation of RWE is necessary to detect potential rare or delayed AEs, further solidifying the evidence base.

### Discrepancies in drug delivery systems and formulations

Beyond the active molecule, biosimilars may exhibit subtle variations in formulation, excipients, and delivery devices compared to the reference biologic. From an antibody engineering perspective, differences in buffer systems or stabilizers can influence protein stability and aggregation propensity, which are critical for minimizing immunogenicity. Furthermore, variations in device design (e.g., PFS) can impact usability and dosing precision. Future developments may see biosimilars integrated into advanced delivery platforms—such as hydrogels or port delivery systems—to extend therapeutic duration and reduce the burden of frequent intravitreal injections [91].

## Limitations

This narrative review has several limitations that should be acknowledged. First, the included studies exhibit substantial heterogeneity in study design, patient populations, outcome measures, and follow-up duration, precluding formal meta-analytic synthesis. Second, the majority of Phase III equivalence trials discussed are industry-sponsored, with primary endpoints assessed at relatively short time points (e.g., Week 8 BCVA), which may not capture long-term differences in efficacy, durability, or safety. Third, long-term safety data beyond one year remain limited for most biosimilars, particularly the more recently approved aflibercept biosimilars. Fourth, no head-to-head biosimilar-versus-biosimilar trials have been conducted, limiting direct comparison between individual products. Fifth, pivotal trials have predominantly focused on nAMD and DME, with under-representation of other approved indications such as RVO, myopic CNV, and PCV. Sixth, the rapidly evolving regulatory and competitive landscape may alter the pharmacoeconomic context described herein, and variations in local regulatory requirements, pricing, and reimbursement structures across jurisdictions further limit the generalizability of cost-effectiveness conclusions.

## Conclusion and future perspectives

Biotherapeutics have revolutionized the management of retinal vascular diseases, yet their high acquisition costs remain a significant barrier to global accessibility. The advent of ophthalmic biosimilars represents a pivotal shift in this landscape. Rigorous regulatory frameworks ensure that these agents demonstrate high structural and functional similarity to originators, with extensive phase III trials and real-world evidence confirming comparable clinical efficacy, safety, and immunogenicity profiles.

By fostering market competition, biosimilars have successfully reduced the economic burden on healthcare systems. However, realizing their full potential requires overcoming implementation barriers, including physician hesitancy regarding indication extrapolation and the need for robust patient education to mitigate nocebo effects. Future research should prioritize long-term pharmacovigilance, particularly for newer aflibercept biosimilars, and explore the integration of these agents with innovative, long-acting delivery technologies. Ultimately, ophthalmic biosimilars stand as a landmark advancement in biopharmaceuticals, offering a sustainable pathway to preserve vision for a broader global population.

## Data Availability

Data sharing is not applicable to this article as no new data were created or analyzed in this study.

## Abbreviations

ADA: Anti-drug antibody
AE: Adverse event
AMD: Age-related macular degeneration
ASP: Average sales price
BCVA: Best-corrected visual acuity
CMC: Chemistry, manufacturing, and controls
CMT: Central macular thickness
CNV: Choroidal neovascularization
CST: Central subfield thickness
CVD: Choroidal vascular disease
DCGI: Drugs Controller General of India
DME: Diabetic macular edema
DR: Diabetic retinopathy
EMA: European Medicines Agency
ETDRS: Early Treatment Diabetic Retinopathy Study
FDA: Food and Drug Administration (United States)
IOI: Intraocular inflammation
mCNV: Myopic choroidal neovascularization
MFDS: Ministry of Food and Drug Safety (South Korea)
MHLW: Ministry of Health, Labour and Welfare (Japan)
MHRA: Medicines and Healthcare products Regulatory Agency (United Kingdom)
nAMD: Neovascular age-related macular degeneration
NMPA: National Medical Products Administration (China)
PCV: Polypoidal choroidal vasculopathy
PD: Pharmacodynamics
PDP: Partnership for Productive Development (Brazil)
PDR: Proliferative diabetic retinopathy
PFS: Prefilled syringe
PK: Pharmacokinetics
PlGF: Placental growth factor
QALY: Quality-adjusted life year
RVD: Retinal vascular disease
RVO: Retinal vein occlusion
RWE: Real-world evidence
SAE: Serious adverse event
TEAE: Treatment-emergent adverse event
VEGF: Vascular endothelial growth factor
WHO: World Health Organization
